# Indoor residual spraying with micro-encapsulated pirimiphos-methyl (Actellic^®^ 300CS) against malaria vectors in the Lake Victoria basin, Tanzania

**DOI:** 10.1371/journal.pone.0176982

**Published:** 2017-05-10

**Authors:** Fabian M. Mashauri, Alphaxard Manjurano, Safari Kinung’hi, Jackline Martine, Eric Lyimo, Coleman Kishamawe, Chacha Ndege, Mahdi M. Ramsan, Adeline Chan, Charles D. Mwalimu, John Changalucha, Stephen Magesa

**Affiliations:** 1 National Institute for Medical Research, Mwanza Centre, Mwanza, Tanzania; 2 Research Triangle Institute (RTI) International, Dar es salaam, Tanzania; 3 Centers for Disease Control and Prevention, Atlanta, United States of America; 4 National Malaria Control Program, Ministry of Health, Community Development, Gender, Elderly and Children, Dar es salaam, Tanzania; Johns Hopkins University, UNITED STATES

## Abstract

**Background:**

The indoor residual spraying programme for malaria vectors control was implemented in four districts of the Lake Victoria basin of Tanzania namely Ukerewe, Sengerema, Rorya andSerengeti. Entomological monitoring activities were implemented in one sentinel village in each district to evaluate the efficacy of pirimiphos-methyl 300 CS sprayed on different wall surfaces and its impact against malaria vectors post-IRS intervention.

**Methods:**

The residual decay rate of p-methyl 300 CS applied at a target dosage of 1g a.i./m^2^ on thesprayed wall surfaces was monitored for a period of 43 weeks post-IRSusing the WHO cone wall bioassay method. The bioassays were performed by exposing 2–5 days old unfed susceptible female *Anopheles gambiae s*.*s*. (Kisumu strain) to sprayed wall surfaces for a period of 30 minutes. In each sentinel village, mosquito collection was carried out by trained community mosquito collectors. Monthly mosquito collections were carried out from 6.00pm to 6.00am using CDC light traps and clay pot methods for indoors host seekingand outdoors resting mosquitoes respectively. Six traps (2 CDC light traps and 4 clay pots) were set per sentinel village per night for28 consecutive days in a moon. PCR and ELISA were used for mosquito species identification and sporozoite detection, respectively.

**Results:**

Based on the WHOPES recommendation, insecticides should have a minimum efficacy of ≥ 80% mosquito mortality at 24 hours post exposure on the sprayed wall surfaces to be considered effective. In this study, p-methyl 300 CS was demonstrated to have a long residual efficacy of 21–43 weeks post-IRS on mud, cement, painted and wood wall surfaces. Numberof anopheline mosquitoes decreased post-IRS interventions in all sentinel villages. The highest numbers ofanopheline mosquitoes were collected in November-December, 38–43 weeks post-IRS. A total of 270 female anopheline mosquitoes were analyzed by PCR; out of which 236 (87.4%) were An. *gambiae* s.l. and 34 (12.6%) were *An*. *funestus* group. Of the 236 *An*. *gambiae s*.*l*.identified 12.6% (n = 34) were *An*. *gambiae s*.*s*. and 68.6% (n = 162) were *An*. *arabiensis*. Ofthe 34 *An*. *funestus* group indentified 91.2% (n = 31) were *An*. *parensis* and 8.8% (n = 3) were *An*. *rivulorum*. The overall *Plasmodium falciparum* sporozoite rate was 0.7% (n = 2,098).

**Conclusions:**

Pirimiphos-methyl 300 CS was found to be effective for IRS in the Lake Victoria basin,Tanzania. P-methyl 300 CShas a long residual efficacy on sprayed wall surfaces and therefore it is effective in controlling principal malaria vectors of *An*. *gambiae* s.l and *An*. *funestus* which rest on wall surfaces after and before feeding.

## Background

Malaria is still a leading cause of morbidity and mortality in sub-Saharan Africa (SSA). It is also the number one public health problem in Tanzania, representing more than 30% of the national disease burden [1]. The decrease in malaria incidence and prevalence has regularly been ascribed to the universal coverage of long-lasting insecticide treated nets (LLINs) campaigns [2, 3], scale-up of indoor residual spraying (IRS) intervention[4, 5]and improved malaria diagnosis and treatment [6–8]. Besides, this decrease has also been observed in areas with restricted or no malaria interventions[9–11]. Furthermore, some studies have recommended that the decrease in malaria vector densities is also a contributing factor towards the decrease in malaria transmission intensity reported in many parts of SSA [12].

Lake Victoria basin region of Tanzania is characterized by highland with unstable malaria transmission prone to malaria epidemics and lowland with stable malaria transmission that act as reservoirs for the highland communities [13]. This region experiences tropical climate with two annual rainy seasons. The short rainy season runs from October to December while the long rainy season is from March to May. However, the length of these seasons may slightly vary from one region to another. Malaria transmission intensity in Lake Victoria basin follows this rainy seasonal cycle with peaks in malaria incidence occurring one to two months following the month of most elevated precipitation [14]. The population of Lake Victoria basin experiences a high malaria prevalence of about 40% [15]. *Plasmodium falciparum* contributes to more than 90% of malaria infections in this region [13]. Three major species of malaria vectors namely *An*. *arabiensis*, *An*. *gambiae s*.*s*. and *An*. *funestus s*.*s* have been previously identified in the Lake Victoria basin [16–18].

Long-lasting insecticide treated nets and insecticides-IRS are the fundamental interventions of present malaria vectors control [19, 20]. While IRS acts by killing blood-fed malaria vectors that might be carrying malaria parasite and hence provides protection to the extensive communities at risk, LLINs principally provide individual and a community protection as they kill host-seeking malaria vectors[21]. Indoor residual spraying utilizing dichloro-diphenyl-trichloroethane (DDT)was the pillar of the worldwide malaria vector eradication campaigns from 1955–1969[22, 23]and was among the World Health Organization’s (WHO) recommended insecticide of choice [24]. However, due to DDT resistant to malaria vectors, alternative insecticides have been experimented in field trials to replace DDT[25]. In addition, the spread of resistance to pyrethroids used in LLINs poseda great concern to malaria vector control interventions [26, 27]. Other alternatives to DDT and pyrethroids resulted from field trials were carbamates and organophosphates. However, these insecticides have short life span on sprayed wall surfaces. For this reason new insecticide formulation with long life span on sprayed wall surfaces were immediately required. A promising technique was the repurposing of existing insecticides not presently utilized in malaria vectors control, together with the advancement of enhanced long life span formulations utilizing micro-encapsulation. A noticeable insecticide candidate coming from this technique was micro-encapsulated pirimiphos-methyl 300 CS (p-methyl 300 CS)[28–30].

Pirimiphos-methyl is an organophosphate which in early field trials of an emulsifiable concentrates (EC) formulation exhibited high level of toxicity, however short life span efficacy against malaria vectors and non malaria vector mosquitoes [31–33]. Syngenta (Basel, Switzerland), the producer of p-methyl, has recently produced micro-encapsulated formulations (CS) with a long lasting efficacy in response to the need to control DDT and pyrethroid resistant malaria vectors [28]. Indoor residual spraying randomized control trials conducted since 1970s indicated high toxicity of p-methyl to malaria vector mosquitoes [34]. Based on these trials, WHO Pesticides Evaluation Scheme (WHOPES) has recommended IRS with p-methyl for a wide use for malaria vectors control as an option control of DDT and pyrethroid resistant malaria vectors.

The IRS programme reported in this paper was implemented in four sentinel districts of Lake Victoria basin, Tanzania in 2014. These districts are namely Ukerewe, Sengerema, Rorya and Serengeti. With the financial support from the U.S. President's Malaria Initiative (PMI) through Research Triangle Institute (RTI) International Tanzania, the National Institute for Medical Research (NIMR), Mwanza Centre, carried outlongitudinal entomological monitoring activities inthesprayed four districts post-IRS intervention. The entomological monitoring activities were performed to evaluate the biological efficacy of p-methyl300 CS on different sprayedwall surfaces and its entomological impact against malaria vectors post-IRS intervention in Lake Victoria basin, Tanzania.

## Methods

### Study areas and population

**Fig 1:**
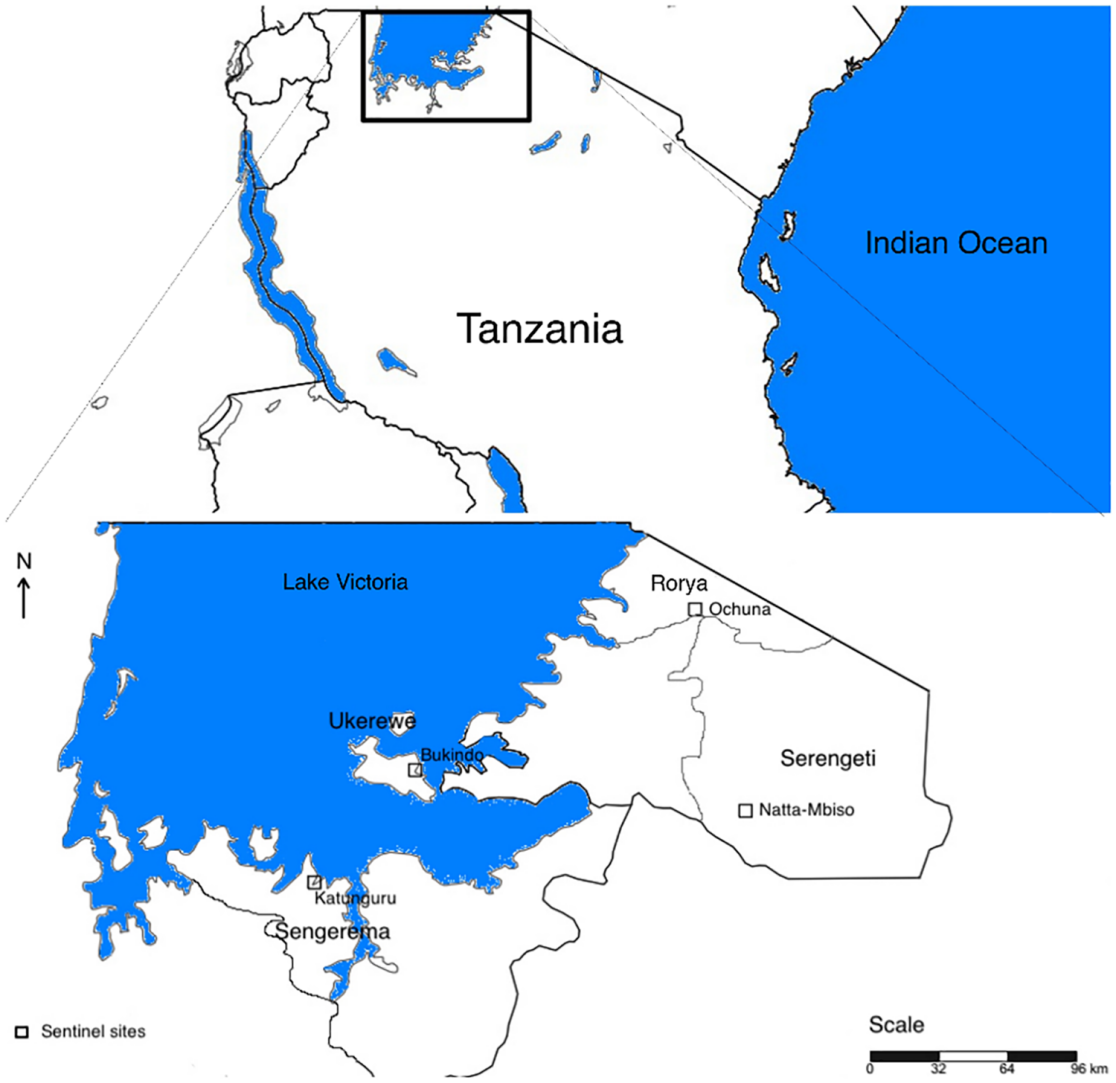
The map of Lake Victoria basin showing the sentinel sites under 2014 IRS campaigns. This map was obtained by manual digitizing via public domain of the Geographic information system (http://www.simplemappr.net/).

Lake Victoria basin is situated in the North-western part of the United Republic of Tanzania (URT), between longitudes 29°E—41°E and latitudes 1°S—12°S. The United Republic of Tanzania occupies an area of 33,756 km^2^ of the Lake Victoria (49% of the total lake surface). The 2014 IRS campaigns were implemented in two regions of Lake Victoria basin, namely Mwanza and Mara. Four districts under IRS campaigns were selected for longitudinal entomological monitoring and assessment of p-methyl 300 CS decay rate using WHO cone wall bioassay tests. The selected districts were Ukerewe and Sengerema for Mwanza region and, Rorya and Serengeti for Mara region (Fig 1). According to the 2012 national census [35] the Mwanza and Mara regions have a total population of 4,516,339. According to Tanzania HIV/AIDS and Malaria Indicator Survey (THMIS) 2012[36], malaria prevalence in under five children in Mwanza andMara regions are 31.4% and 30.3% respectively. The Lake Victoria basin regions have a higher malaria prevalence compared to other regions in the country. Lake Victoria basin experiences tropical climate with two annual rainy seasons. The short rains occur yearly from October to December and the long rain season happen yearly from March to May, but the length of rain and dry seasons may slightly vary from one region to another. Indoor residual spraying campaign and monitoring only covers a single of these potential malaria transmission peaks in this study area (Fig 1).

### Indoor residual spraying campaigns 2014

Pirimiphos-methyl300 CS was selected for spraying on wall surfaces for 2014 IRS campaigns in Ukerewe, Sengerema, Rorya and Serengeti districts. P-methyl300 CS was selected as an alternative insecticide of rotation followingthe previous2012-13 IRS campaignswhere deltamethrine K-Othrine (WG 250, Bayer) was sprayed in Sengerema, Rorya and Serengeti districts and bendiocarb (FICAM 80% WP, Bayer) wassprayed in Ukerewe district (RTI-International unpublished report). The 15-liter-capacity H. D. Hudson Manufacturing Company (Chicago, IL, USA) 67422 AD, Hudson^®^ X-pert spray pumps, recommended by the WHO for use in IRS were used. One bottle containing 833ml of p-methyl 300CS was mixed in 10 liters of clean water in the spray can and pressurized to 55 Psi as per manufacturers’ instruction. The 10 litersof insecticide mixture was applied over a 250m^2^ resulting in a target dosage of one gram of active ingredient per square metre (1g a.i. /m^2^). Only one round of IRS was carried out in the beginning of the long rainy season in March 2014(Table 1).

**Table 1:** Spraying schedule in districts and timing of first monitoring visit post IRS.

S/N	District	Village	Date of spray	Insecticide sprayed	Weeks post-IRS
**1**	Ukerewe	Bukindo	15/03/2014	P-methyl 300 CS	2
**2**	Sengerema	Katunguru	15/03/2014	P-methyl 300 CS	3
**3**	Rorya	Ochuna	25/03/2014	P-methyl 300 CS	1
**4**	Serengeti	Natta-Mbiso	20/03/2014	P-methyl 300 CS	1

The IRS operation was performed by volunteers selected from the local communities who were trained by RTI international staff whereas the IRS coordination was done by District Health Management Team through a dedicated team of District IRS Technical Team (DITT). Structure coverage with IRS was scored at > 80% in all sprayed districts.

### Mosquito collection, processing and identification

In each district, twenty four houses with different building materials were selectedper sentinel village for longitudinal entomological monitoring. Mosquito collection was carried out by trained community mosquito collectors (CMCs) under the supervision of trained District Vector Control Officers(DVCOs). Monthly mosquito collections were carried out from 6.00pm to 6.00am using standard Centre for Diseases Control (CDC) light traps (CDC, Atlanta, GA, USA) and clay pot method for indoor host seeking and outdoors resting mosquitoes, respectively. Collected mosquitoes were identified by CMCs based on morphological characteristics. Likewise all collected mosquitoes were sorted by sex using the keys described by Gillies and Coetzee[37]. Mosquitoes from the sentinel villages were stored in vials with silica gel and transported once every month to the NIMR Mwanza central laboratory for molecular analysis.

### CDC Light trap method

In each selected sentinel village, twenty four houses were conveniently selected for mosquito collection using CDC light traps. Two CDC light traps (one CDC light trap per house) were set in two houses per night. Mosquitoes were collected for28 consecutive days in a moon in rotation. Briefly, in the selected houses the CDC light trap was installed at about 1.5m above the floor next to the head of the sleeping person(s). The person(s) was requested to sleep under an untreated mosquito net(s) overnight. CDC light traps were set to operate from 6.00pm to 6.00am of the next morning to trap mosquitoes seeking for hosts. Captured mosquitoes were transferred separately into labeled paper cups covered with a piece of netting material.

### Clay pot method

The clay pot method was used to collect outdoor resting mosquitoes. The pots were molded by local potters using clay soil available from the sentinel village. The clay pots were made of size 0.5m diameter with an opening of 20cm width. Each clay pot had a 2cm hole made at the bottom of the pot rendering them useless for storage of water as they allowed water to freely drain out. In each selected sentinel village, twenty four houses were selected for clay pots traps. Two houses per night were used to set up four clay pots traps (i.e. 2 clay pots per house) outdoors overnight, near selected houses with different construction materials. The pots were set up from 6.00pm to 6.00am of the next morning. Clay pots were set for 28 consecutive days in rotation. The pots were positioned at an inclined angle to let mosquitoes enter and rest inside the dark inner wall surface of the pot. In the morning at 06.00am, the CMCs covered the opening using a piece of netting fabric with a small entry hole for inserting an aspirator to suck out mosquitoes and transfer them into a labeled paper cup.

### Rearing of susceptible mosquito colony

Susceptible colony of *Anopheles gambiae s*.*s* (Kisumu strain) was reared at NIMR Mwanza insectary. Mosquitoes were maintained under a controlled condition in the insectary as described by WHO[38]. The reared mosquitoes were used for cone wall bioassay tests to evaluate the efficacyof p-methyl 300 CS on different sprayed wall surfaces.

### Bio-efficacy of p-methyl 300 CS on sprayed wall surfaces

The residual decay rate of p-methyl300 CS using a target dosage of1g a.i./m^2^ on thesprayed wall surfacewas monitored for a period of 43 weeksusing WHO cone tests[39]. Throughout the monitoring period, one village from each of the 4 districts was sampled for residual insecticide decay rate bioassay tests, using limited random sampling of villages within a 20km radius of the district medical office. Bioassays were carried out in the following villages in Mwanza region: Katunguru in Sengerema district, and Bukindo in Ukerewe district. In Mara region the following villages were sampled: Ochuna in Rorya district, and Natta-Mbisso in Serengeti district (Fig 1). To minimize potential bias that may arise, houses sprayed by different spray operators, spray teams and with different wall substrates were selected for testing; one house of each wall surface type commonly found in the area were randomly selected for cone bioassay in each sentinel site for the monitoring. The most common wall surface finishing found in the selected sites was mud, cement, wood, whitewash and burnt brick. Furthermore, in each house, one room (living room) was tested. To monitor the insecticide decay rate on the different wall surfaces in each room, 3 walls of the room were tested(i.e. upper, middle and lower positions), by fixing each of the cones at about 1.0m, 1.5m and 2.0m high on each wall. Three cone assays were carried out in any one house using 20 unfed susceptible adult female *Anopheles gambiae*(Kisumu strain) mosquitoes per cone. One replicate of cone was used per unsprayed block surface (control). The bioassays were performed by exposing 2–5 days old *An*. *gambiae s*.*s*. (kisumu strain) for a period of 30 minutes. The *An*. *gambiae s*.*s*. were exposed on sprayed wall surfaces and on an unsprayed block surface (control) as described by WHO[39, 40]. Following exposure, the *An*. *gambiaes*.*s* were placed in 150ml plastic cups (1 replicate per cup) with sucrose solution provided and maintained in a cool box for 24hours at 27°C ±2°C and 80% ±10% RH. Percentage of knockdown (KD) after 60minutesand percentage mortality after 24hours was scored.

### Correction of mortality in bioassays

Test series with control mortality of over 20% were cancelled. Those with control mortality between 5% and 20% were corrected by Abbott’s formula (1925)[41]as follows:

#### Abbott's formula

$$Corrected\;\%=\left(1-\frac{n\;in\;T\;after\;treatment}{n\;in\;Co\;after\;treatment}\right)*100$$

Where n = number of mosquitoes died after 24 hours, T = treated wall bioassay, Co = control bioassay

### Laboratory processing

#### (i) Circumsporozoite Enzyme-linked Immunosorbent Assay (ELISA) for detection of malaria sporozoite

The head and thorax of individual female anophelinemosquitoes were homogenized in 250uL of grinding buffer(PBS, pH 7.4 containing 0.4% 0.1N NaOH and 0.5% casein)using a plastic pestle. CS protein micro-plate ELISA using 50uL/well of the homogenate was done in 96-well microtitre plates coated with anti-*P*. *falciparum* and anti-*P*.*vivax* monoclonal antibodies at 22–25°C for 30min[42]. Captured CS antigen was revealed by monoclonal antibody (MoAb) horseradish peroxidase conjugate incubated for 1hr. Addition of ABTS [2,2^'^-azino-di-(3-ethylbenzthiazoline-6-sulphonate)] substrate gave a blue-greenish colour reaction for positive results which were read by visual assessment of the colour reactions, and OD measured within 30min using spectrophotometer (MRX Dynex Technologies, Virginia, Chantilly, USA) at 450nm. Sample positivity was determined by calculating a cut-off value from the negative control OD value i.e any value above the twice average value of seven negative controls was positive.

#### (ii) Polymerase Chain Reaction (PCR) for mosquito species identifications

PCR was carried out using a modified method of Scott *et al* protocol as described elsewhere[43]. Briefly, 1μL of DNA extracted from a mosquito, 5μL of 5x Go Taq PCR reaction buffer with MgCl_2_, 2.5μL of 2.5mM of each dNTP, 1μL of 25mM MgCl2, 0.15uL of 5 units/μL of Taq DNA polymerase, 1μL of each 25pmol/μL of primers UN, AR, GA and ME, 2μL of each 25pmol/μL of primers QD and QDA, and sufficient sterile water to give a total volume of 25μL. The PCR was carried out with an initial denaturation at 95°C for 5min followed with a program of 30 cycles of denaturation at 95°C for 30sec, annealing at 50°C for 30sec, and extension at 72°C for 30sec, and final extension of 72°C for 5min. After the PCR was completed, the 8μL reaction volume was mixed with a standard 2μL agarose gel loading buffer containing a small amount of bromophenol blue, and electrophoresed through a 2% agarose-Tris-borate-EDTA gel containing Red Safe DNA Stain (Promega, Madison, USA). The amplified fragments were visualized by illumination with short wave ultraviolet light.

### Data analysis

The data were double-entered, verified, validated and checked for consistency using excel. Then cleaned dataset was transferred into STATA (Version.12, College Station, Texas, USA). The mosquito data from the houses were summarized per 28 night trap days in a month. The outcomes of interest were the proportions of each species trapped in a month. Percentage mortalities at 24hrs post-exposure were used to assess the efficacy of the p-methyl 300 CS according to WHO criteria[44]. Regression curves that show the rate of insecticide decay rate versus number of weeks post spraying was used for each sprayedwall surface. The criteria of using regression curves was to keep consistency with other previously published work[45]. Abbot’s formula[46] was used for control mortality correction between 5%-20%. Furthermore, sporozoite rate and mosquito species were determined as proportional percentage from each sentinel village.

### Ethical considerations

This study was undertaken as part of the ongoing national malaria programme aimed to control malaria through IRS around Lake Victoria Victoria basin. The ethical approval for this study was granted by the Lake Zone Institutional Review Board (LZIRB) of the National Institute for Medical Research (NIMR), Tanzania. The informed consent was sought verbally from head of households to use their houses for bioassays and mosquitoes collection. In addition, the permission to carry out IRS monitoring was obtained from District Medical Officers (DMOs) and village leaders.

## Results

### IRS implementation

The 2014 IRS campaignswere carried out in four districts of Lake Victoria basin of Tanzania namely Ukerewe, Sengerema, Rorya and Serengeti in March 2014 (Table 1). The timing of the IRS campaigns was conducted during the beginning of long rainy season in the basin. P-methyl 300 CS was selected for the 2014 IRS campaigns as an alternative insecticide of rotation after two years of bendiocarb and deltamethrin spraying in the Lake Victoria basin.

### Insecticide residual decay rate

**Fig 2:**
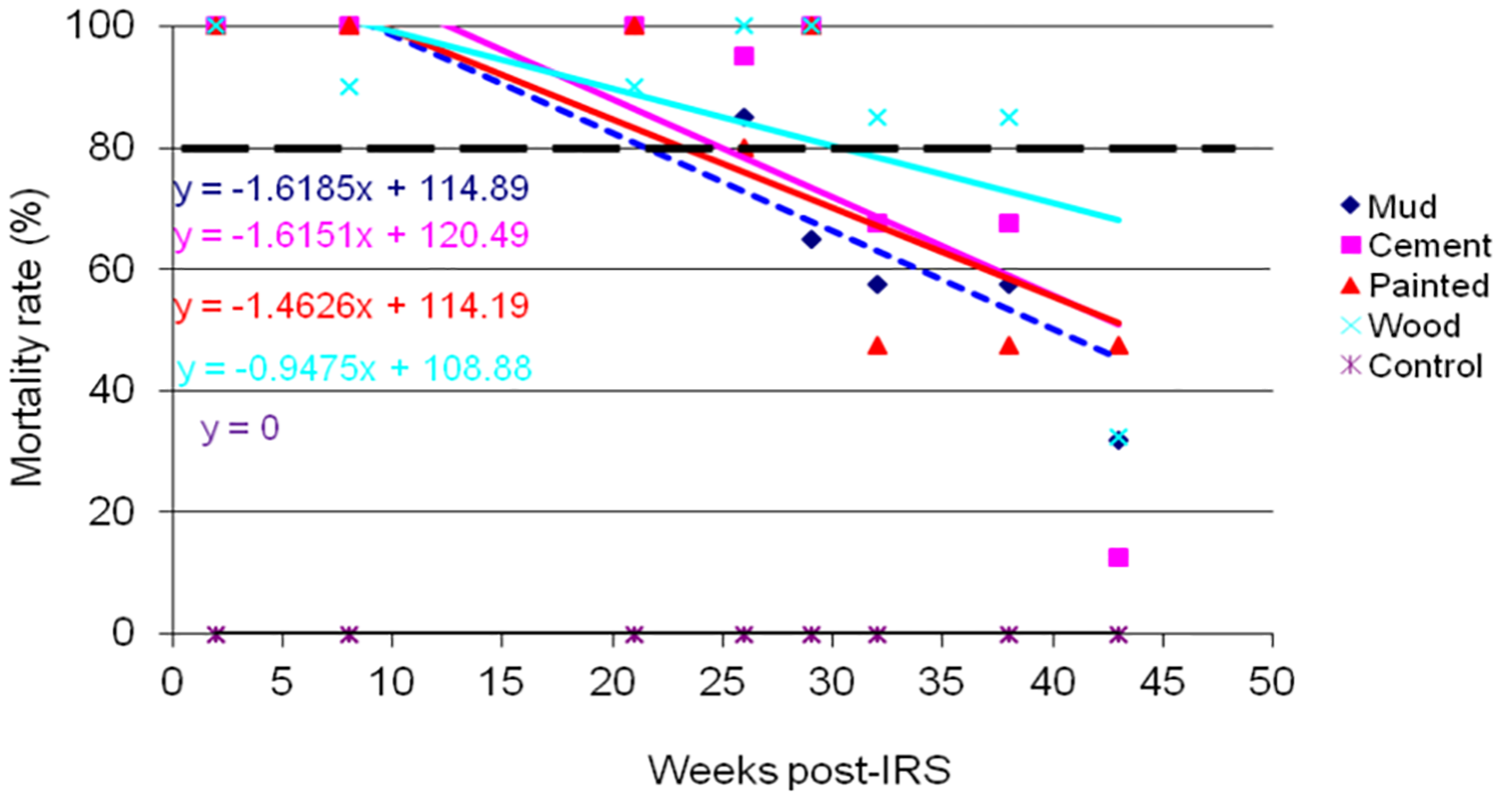
Cone wall bioassay response of susceptible strain *An*. *gambiae s*.*s* to p-methyl 300 CS sprayed in different wall surfaces in Bukindo village, Ukerewe district. The dashed line represents the WHO threshold of 80% mortality.

**Fig 3:**
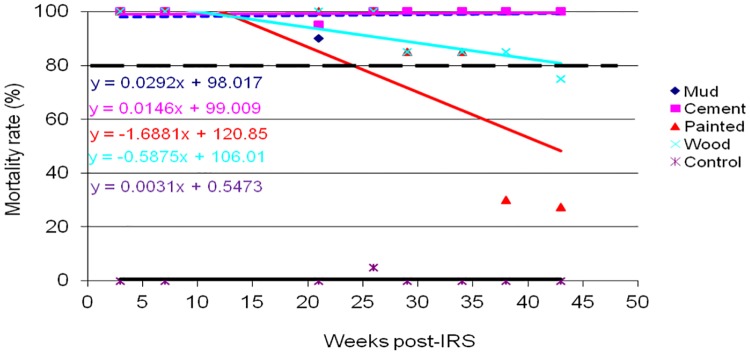
Cone wall bioassay response of susceptible strain *An*. *gambiae s*.*s* to p-methyl 300 CS sprayed in different wall surfaces in Katunguru village, Sengerema district. The dashed line represents the WHO threshold of 80% mortality.

**Fig 4:**
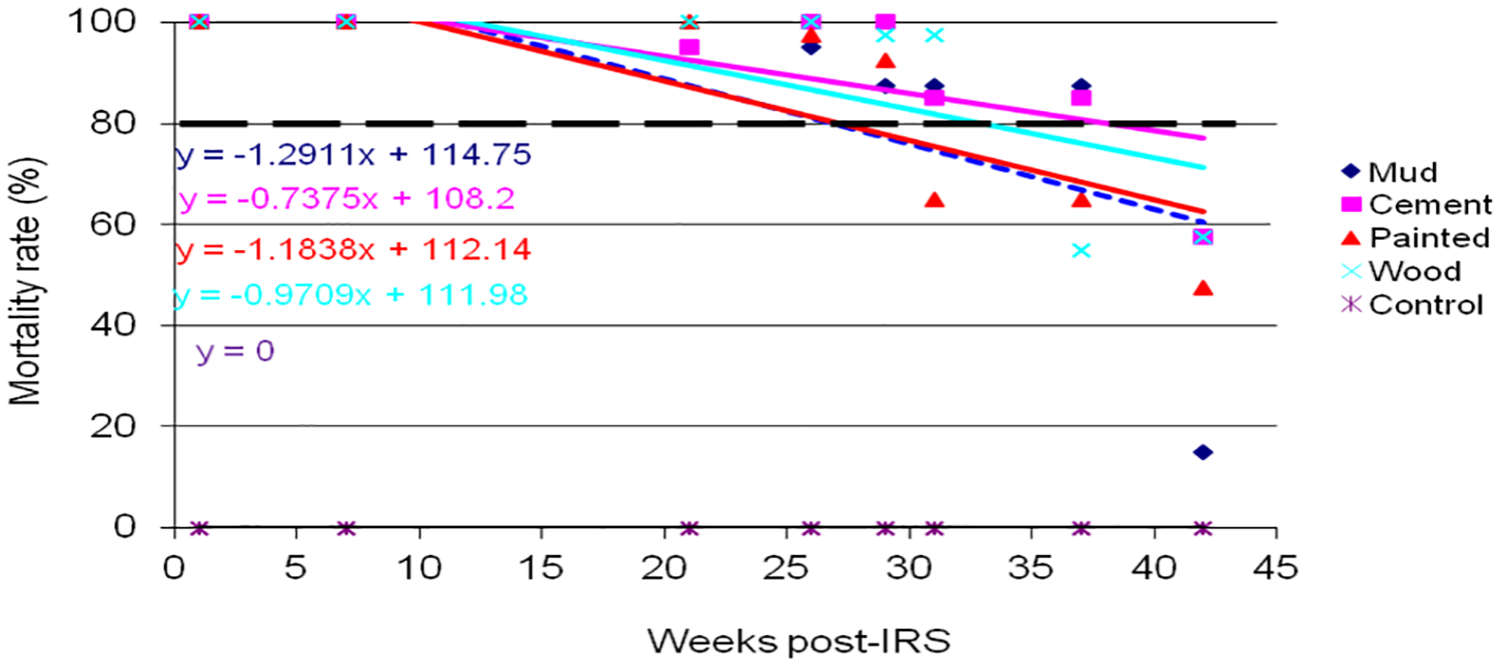
Cone wall bioassay response of susceptible strain *An*. *gambiae s*.*s* to p-methyl 300 CS sprayed in different wall surfaces in Ochuna village, Rorya district. The dashed line represents the WHO threshold of 80% mortality.

**Fig 5:**
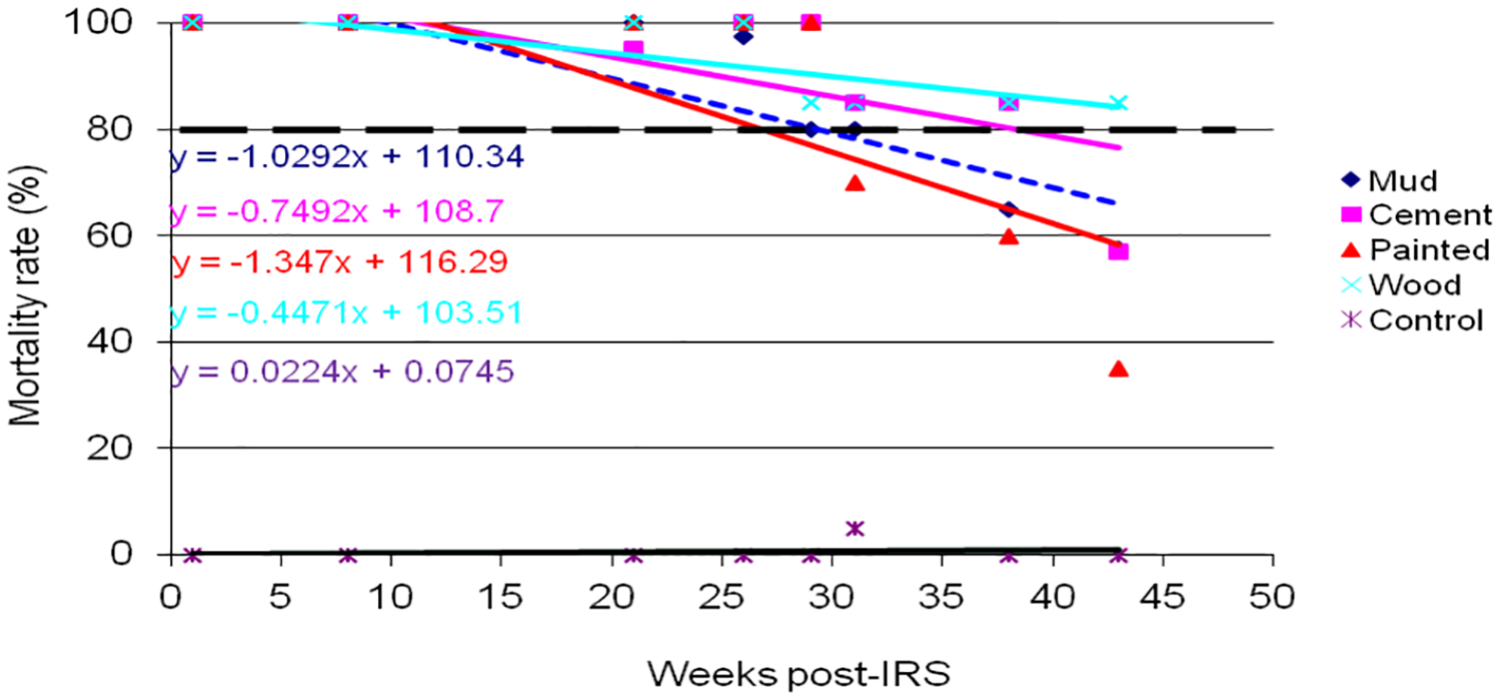
Cone wall bioassay response of susceptible strain *An*. *gambiae s*.*s* to p-methyl 300 CS sprayed in different wall surfaces in Natta-Mbisso village, Serengeti district. The dashed line represents the WHO threshold of 80% mortality.

The results of WHO cone wall bioassays for monitoring p-methyl 300 CS residual decay rate on sprayed wall surfaces of mud, cement, painted and wood are shown in Figs 2–5. *An*. *gambiae s*.*s*. Kisumu strain was exposed on the sprayed wall surfaces over a period of 43 weeks post-IRS monitoring period. The bioassay test was conducted monthly from March to December 2014. Different patterns of p-methyl 300 CS residual efficacy decay rate were observed among the sprayed wall surfaces in sentinel villages as shown in Figs 2–5. The residual decay rate of p-methyl 300 CS are demonstrated using regression curves that show the rate of p-methyl 300 CS decay rate against number of week post-IRS.

In Ukerewe district, wood wall surface retained the WHOPES recommended residual efficacy of ≥ 80% for 32 weeks post-IRS monitoring period. However, cement and painted wall surfaces retained the recommended residual efficacy for 26 weeks post-IRS, while mud wall surface retained the recommended residual efficacy of ≥ 80% for 21 weeks post-IRS before falling below the recommended level (Fig 2).

In Sengerema district; mud, cement and wood wall surfaces retained the WHOPES recommended residual efficacy of ≥ 80% for 43 weeks post IRS. Moreover, painted wall surface retained the recommended residual efficacy of ≥ 80% for 26 weeks post-IRS before falling below the recommended level (Fig 3).

In Rorya district, cement wall surface retained the WHOPES recommended residual efficacy of ≥ 80% for 37 weeks post-IRS monitoring period, while wood wall surface retained the recommended residual efficacy for 31 weeks post-IRS. Furthermore, mud and painted wall surfaces retained the recommended residual efficacy for 29 weeks post-IRS monitoring period before falling below the recommended level (Fig 4).

In Serengeti district, wood wall surface retained the WHOPES recommended residual efficacy of ≥ 80% for 43 weeks post-IRS monitoring period. Cement wall surface retained the recommended residual efficacy of ≥ 80% for 38 weeks, while mud and painted wall surfaces retained the recommended residual efficacy of ≥ 80% for 29 weeks before falling below the recommended level (Fig 5).

### Impact of IRS on mosquito collection in Ukerewe district

**Fig 6:**
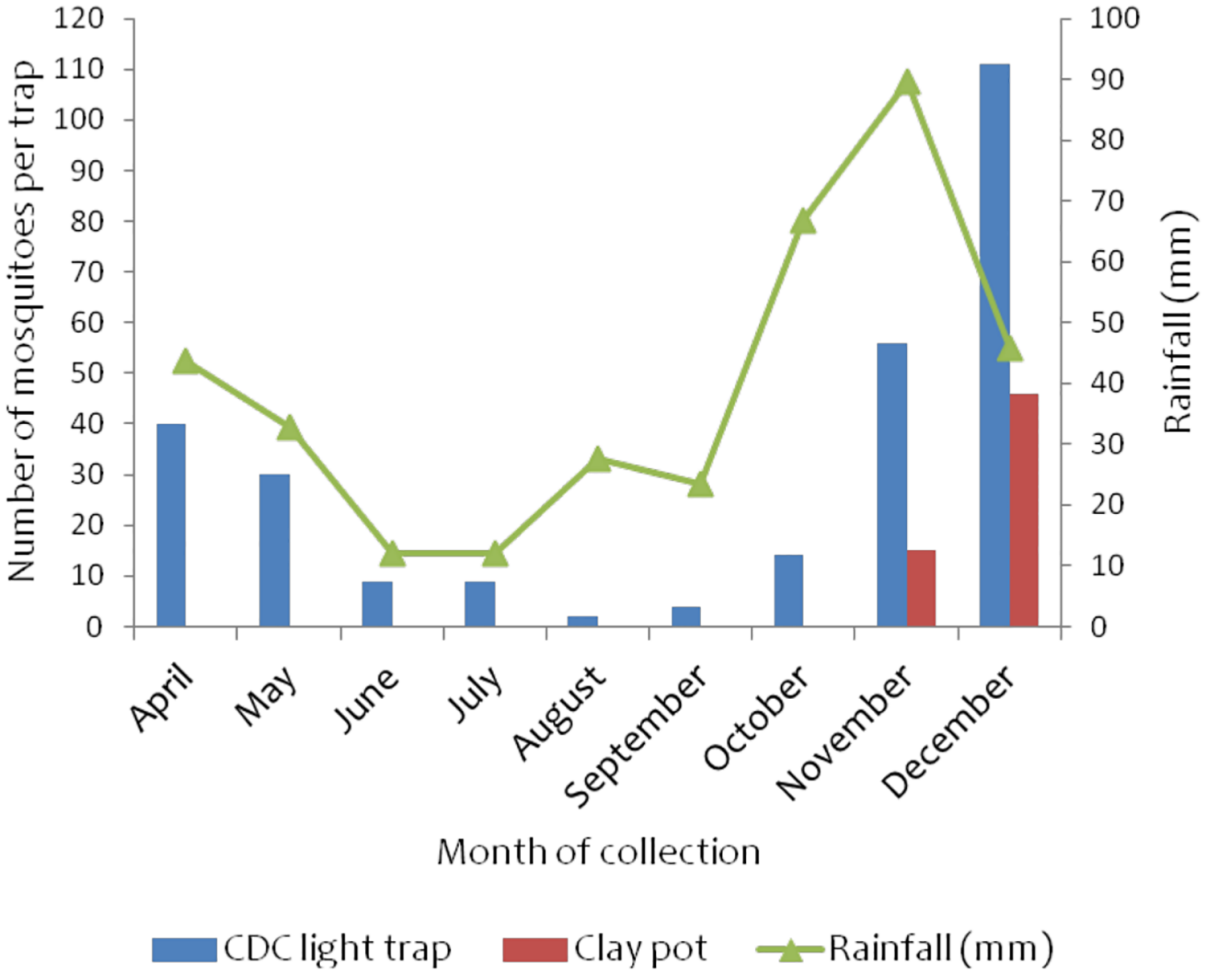
Monthly collection of anopheline mosquitoes in Bukindo village, Ukerewe district monitored using CDC light traps and clay pot methods. The rainfall data was obtained from climate data library (http://iridl.ldeo.columbia.edu/maproom/).

**Fig 7:**
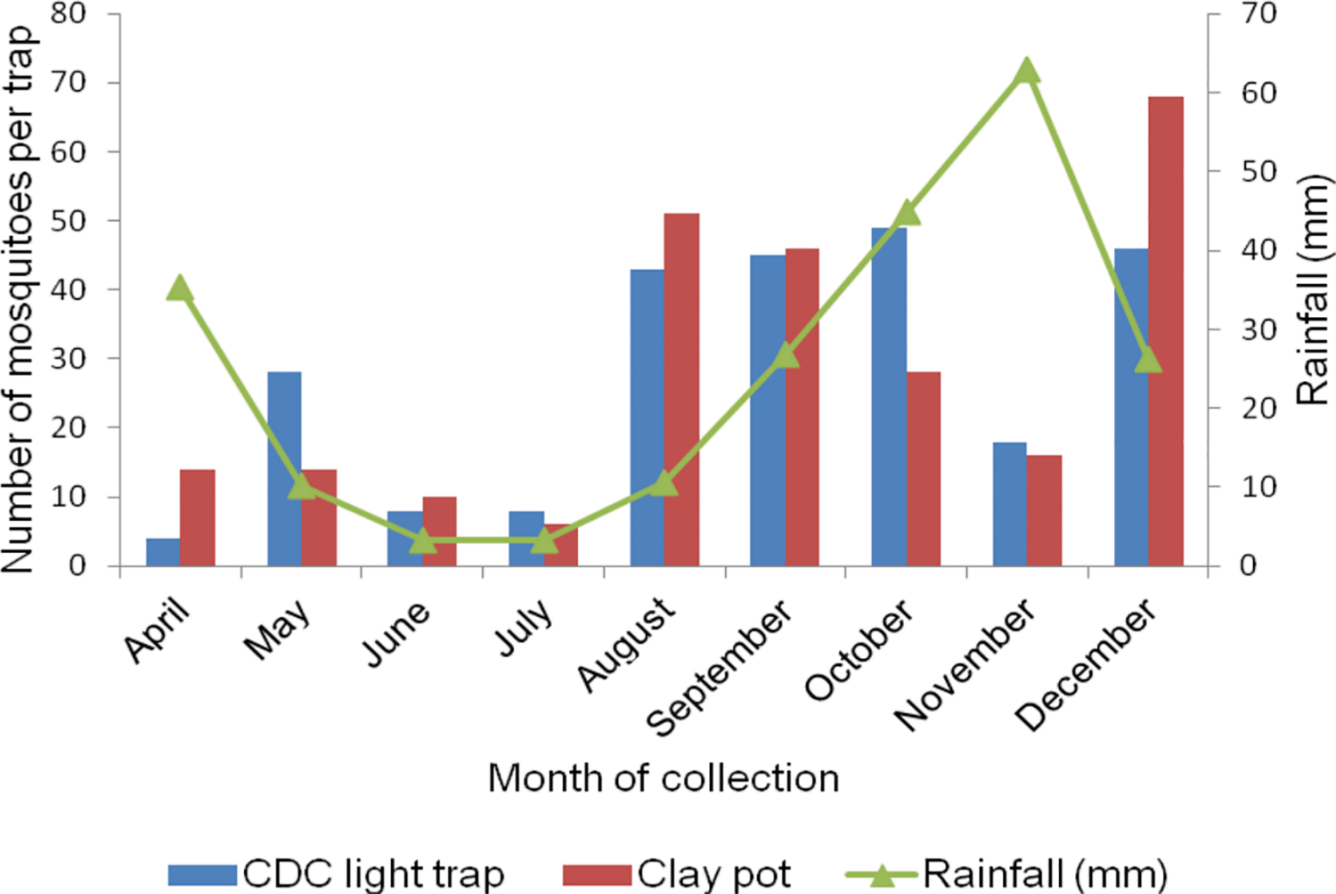
Monthly collection of anopheline mosquitoes in Katunguru village, Sengerema district monitored using CDC light traps and clay pot methods. The rainfall data was obtained from climate data library (http://iridl.ldeo.columbia.edu/maproom/).

**Fig 8:**
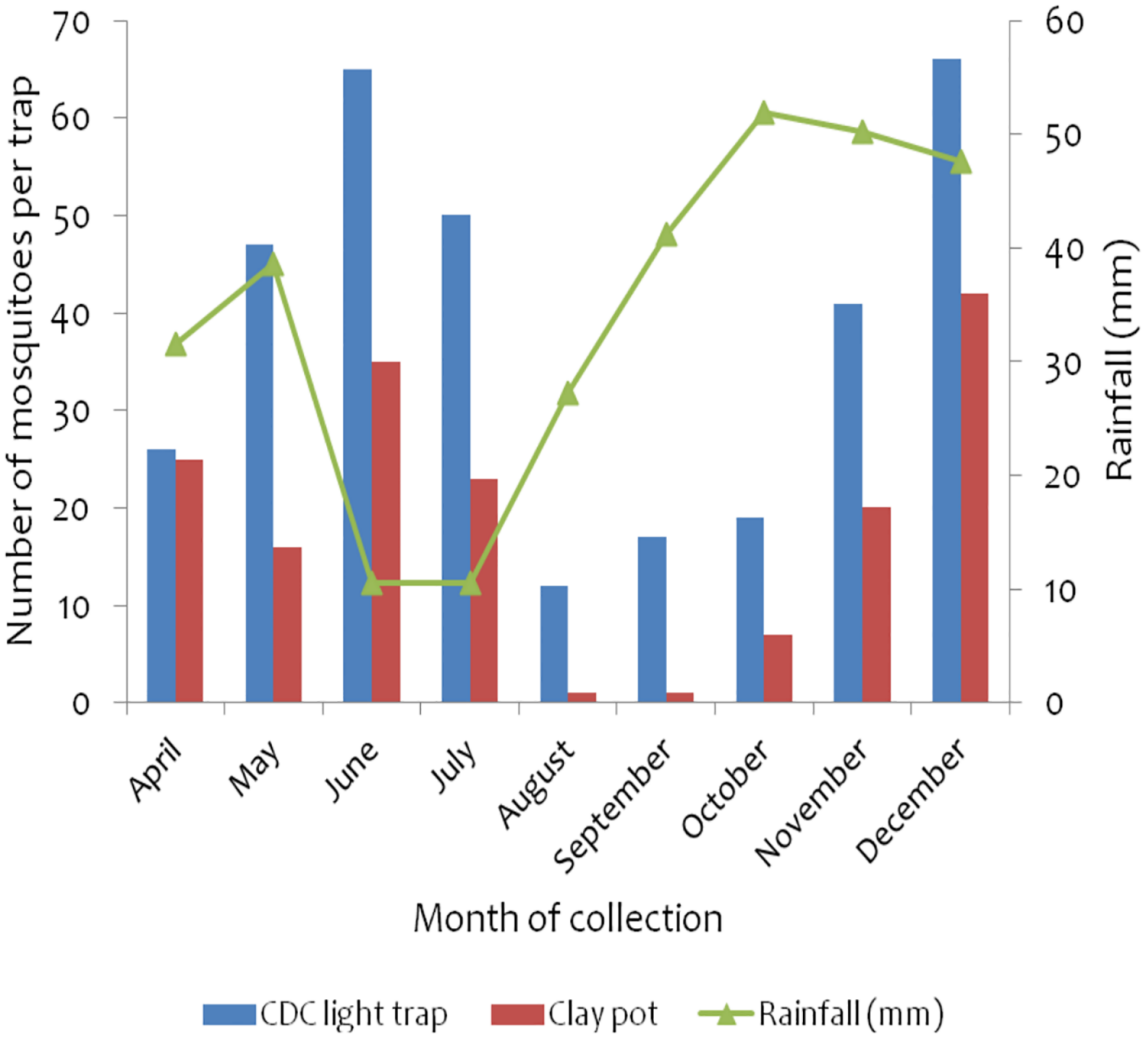
Monthly collection of anopheline mosquitoes in Ochuna village, Rorya district monitored using CDC light traps and clay pot methods. The rainfall data was obtained from climate data library (http://iridl.ldeo.columbia.edu/maproom/).

**Fig 9:**
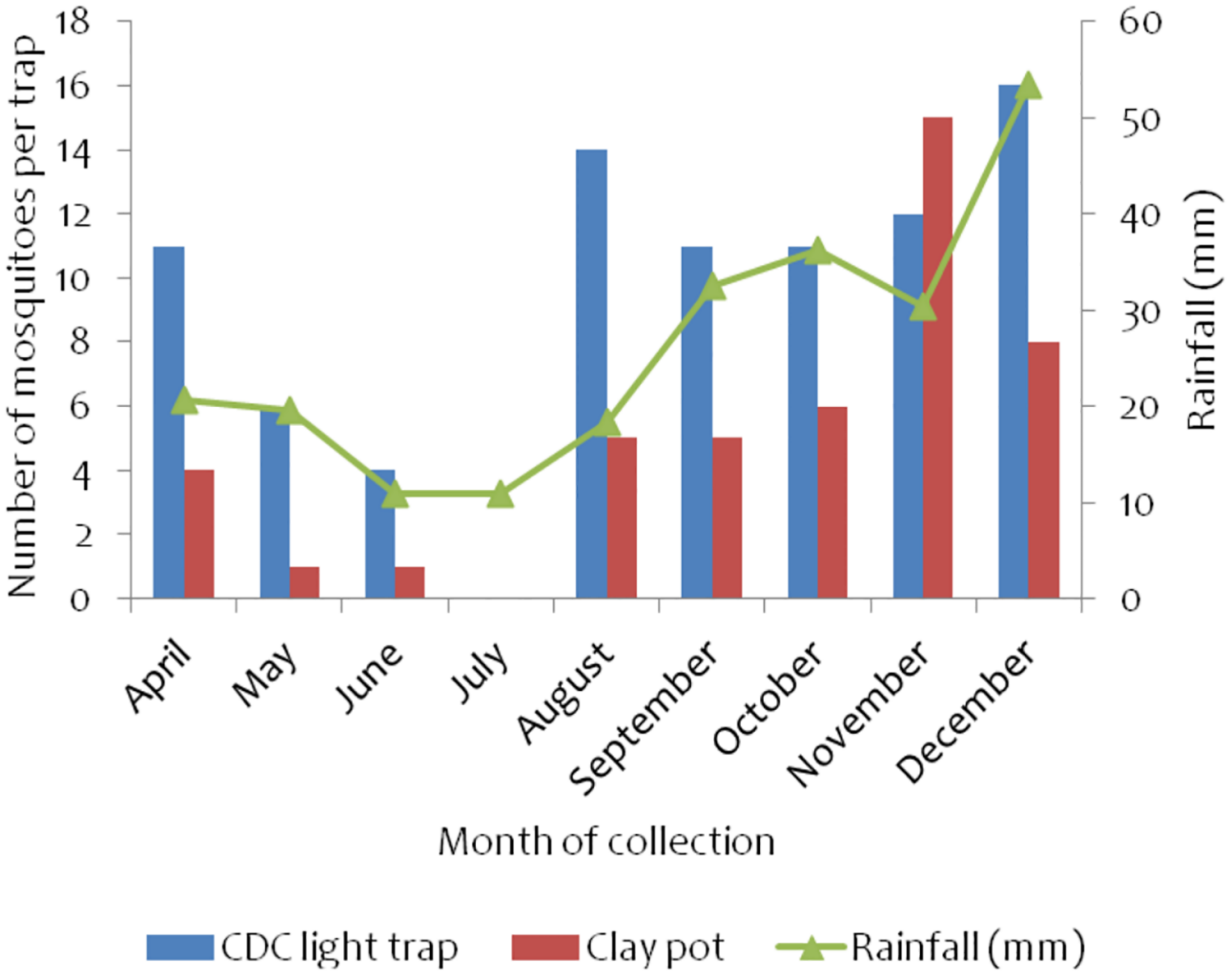
Monthly collection of anopheline mosquitoes in Natta-Mbisso village, Serengeti district monitored using CDC light traps and clay pot methods. The rainfall data was obtained from climate data library (http://iridl.ldeo.columbia.edu/maproom/).

Fig 6: Shows mosquito collection in Bukindo sentinel village, Ukerewedistrict. The bars showstotal number of female anopheline mosquitoes collected per trap per month with each trapping method while the line graph shows the trend of rainfall in each month over the monitoring period. The number of anopheline mosquitoes collected in Figs 6–9 includes both *An*. *gambiae* s.l. complex and *An*. *funestus* group.

Overall, low numbers of anopheline mosquitoes were collected during the monitoring period with the exception of November and December when relatively higher numbers of anopheline mosquitoes were collected (71 and 157 respectively). No anopheline mosquitoes were collected in clay pots from April to October. All mosquitoes were collected by CDC light traps only during the monitoring period with the exception of November and December when both CDC light traps and clay pot methods collected anopheline mosquitoes in the district (Fig 6).

### Impact of IRS on mosquito collection in Sengerema district

Overall, a high numbers of anopheline mosquitoes were collected in August to December (except in November) by CDC light traps and clay pots methods in Katunguru sentinel village in Sengerema district range between 34–114 mosquitoes. There was a gradual increase of anopheline mosquito collection from August to December. An increase in precipitation was subsequently noticed during the same months range between (10.57 mm to 63.2 mm)(Fig 7).

### Impact of IRS on mosquito collection in Rorya district

CDC light traps and clay pot methods were used to collect mosqutoes inOchuna sentinel village in Rorya district. The CDC light traps collected higher number of mosquitoes compared to clay pots methods throughout the monitoring period (343 vs 170). Higher numbers ofanopheline mosquitoes were collected in May to July (range between 63–100)and November to December(61 and 108 respectively) in the district. Higher numbers of anophelines mosquitoes in Rorya district was found to correspond positively with rainy season (Fig 8).

### Impact of IRS on mosquito collection in Serengeti district

In Natta-Mbiso sentinel village in Serengeti district, anopheline mosquito collection decreased gradually from April to June by both collection methods (15 to 5 mosquitoes). There was no anopheline mosquito collected by any collection methods during the month of July. However, anopheline mosquito collection started to increase from August and reached highest numbers in November and December (27 and 24 mosquitoes respectively). Anopheline mosquito abundance was observed to correspond well with the increases in precipitation (Fig 9).

### Anopheline mosquito sporozoite rates

A total of 2,098 of anopheline mosquitoes were tested for the presence of *P*. *falciparum* sporozoites using the CSP-ELISA method. The results on the presence of sporozoites suggest an estimated overall sporozoite rate of 0.7% inthe foursprayed districts. Serengeti district had a relatively higher sporozoite rate estimatedat 1.6%, followed by Ukerewe and Rorya districts with a sporozoite rate estimate of 0.7%, while Sengerema district had a sporozoite rate of 0.6% (Table 2).

**Table 2:** Sporozoite rates of anopheline mosquitoes collected from sentinel sites.

Sentinel site	No. tested (n)	ELISA Pf +ve	Sporozoite rate (%)
Sengerema	537	3	0.6
Ukerewe	874	6	0.7
Rorya	560	4	0.7
Serengeti	127	2	1.6
**Total**	**2098**	**15**	**0.7**

### Anopheline mosquitoe species identification

A total of 270 malaria vector mosquitoes were identified by PCR during entomological monitoring of IRS in Lake Victoria basin, Tanzania. Of the anophelinemosquitoes tested, 236 (87.4%) were identified as *Anopheles gambiae* sensu lato complex and 34 (12.6%) were found to belong to the *An*. *funestus* group. Out of the 236 *An*. *gambiae s*.*l*. samples that were identified by PCR, 74 (31.4%) were identified as *An*. *gambiae s*.*s*. and 162 (68.6%) as An. *arabiensis*. Of 34 *An*. *funestus* group membersidentified further by PCR, 31 (91.2%) were identified as *An*. *parensis* and 3 (8.8%) belonged to *An*. *rivulorum*. Both *An*. *parensis* and *An*. *rivulorum* were collected only in Sengerema district. *An*. *parensis* were collected by both collection methods whereas An. *rivulorum* were collected only by clay pot method (Table 3). *An*. *gambiae* s.s. and *An*. *arabiensis* were the most main species collected by CDC light traps in Ukerewe, Rorya and Serengeti districts. Besides, *An*. *parensis* were the most principal species collected by clay pot method in Sengerema district (Table 3).

**Table 3:** Anopheline mosquito species identification collected by CDC light traps and clay pots method from sentinel sites in Lake Victoria, basin.

District	Collection method	No. sample (N)	*An*. *gambiae* n (%)	*An*. *arabiensis* n (%)	*An*. *funestus* n (%)	*An*. *parensis* n (%)	*An*. *rivulorum* n (%)
**Sengerema**	CDC	21	3(14.3)	4(19)	0(0)	14(66.7)	0
	Clay pot	26	3(11.5)	3(11.5)	0(0)	17(65.4)	3(11.5)
**Ukerewe**	CDC	65	36(55.4)	29(44.6)	0(0)	0(0)	0(0)
	Clay pot	8	8(100)	0(0)	0(0)	0(0)	0(0)
**Rorya**	CDC	71	6(8.5)	65(91.5)	0(0)	0(0)	0(0)
	Clay pot	33	1(3.0)	32(97)	0(0)	0(0)	0(0)
**Serengeti**	CDC	33	13(39.4)	20(60.6)	0(0)	0(0)	0(0)
	Clay pot	13	4(30.8)	9(69.2)	0(0)	0(0)	0(0)
**TOTAL**		270	74(27.4)	162(60)	0(0)	31(11.5)	3(1.1)

## Discussion

The residual efficacy of p-methyl 300 CS at a dosage rate of 1g a.i./m^2^ was evaluated to monitor the p-methyl 300 CS decay rate on sprayed wall surfaces made of mud, cement, painted and wood for a period of 43 weeks post-IRS in all four districts in the Lake Victoria basin regions of Mwanza and Mara, Tanzania. In the Tanzania Lake Victoria basin regions of Mwanza and Mara, mud wall surfaces represent 81%, cement 17% while the other wall surfaces constitutes only 2% of all sprayed wall surfaces [47]. In light of WHOPES recommendation, ideal insecticides should have a minimum efficacy of ≥ 80% mosquito mortality at 24hours, following a 30 minutes exposure on a sprayed wall surface. The pattern of p-methyl 300 CS decay rate on mud, cement, painted and wood wall surfaces reported in this study differ from one sentinel village to another. This survey demonstrates that p-methyl 300 CS residual efficacy on mud wall surfaces were below the WHOPES recommended threshold of ≥ 80% after 21–29 weeks. This observation is in concurrence with the study carried out by Chanda *et al* in Zambia [48]. Cement wall surfaces maintained the residual efficacy of ≥ 80% for a period of 32–43 weeks post-IRS intervention. Nevertheless, painted wall surfaces maintained the residual efficacy of ≥ 80% for 26–43 weeks in all sentinel villages before falling below the WHOPES recommended threshold. Similarly, wood surfaces maintained the residual efficacy of ≥ 80% for a period of 32–43 weeks post-IRS before falling below the recommended threshold. Findings from past studies demonstrated that p-methyl 300 CS rapidly decays and as a result loses efficacy on permeable wall surfaces such as mud while maintaining persistent residual efficacy on less permeable wall surfaces. In this study, p-methyl 300 CS showed persistent longer residual efficacy on cement and wood surfaces in all sentinel villages compared with mud surfaces against a susceptible Kisumu strain of *An*. *gambiae* s.s. This finding is in agreement with results from the study of Haji *et al* [49].

In this study p-methyl 300 CS have demonstrated a longer residual efficacy of 21–43 weeks post IRS on mud, cement, painted and wood wall surfaces. This finding is in agreement with previous studies which shows that p-methyl 300 CS has longer residual efficacy of 20–36 weeks[28, 34, 50]. This long residual efficacy of p-methyl 300 CS on sprayed wall surfaces is sufficient to protect communities for one season of malaria transmission in the Lake Victoria basin, Tanzania [51, 52]. Providing universal coverage of LLINs to populations at risk of malaria hasbecome a priority for many SSA National Malaria Control Programmes (NMCP) in recent years (1). Pyrethroid resistant malaria vectors are now present in SSA, and the frequency of resistance is increasing due to high selection pressure from high LLINs and IRS coverage[53–57]. Long-lasting insecticide treated nets can still provide personal protection through the “mechanical barrier” particularly when the net do not have holes[53]. With the campaigns of universal coverage of LLINs in many parts of SSA and the scaling-up of IRS using p-methyl 300 CS constitutes a potential tool for insecticide resistance management. Randomized control trials have shown that a combined intervention of p-methyl 300 CS IRS and pyrethroid LLINs provides added protection against malaria infection[58]. Recent case control trials of the combination intervention of LLINs and p-methyl 300 CS IRS in experimental huts against pyrethroid resistant *An*. *gambiae* support that hypothesis[21, 59].

The IRS campaigns in Lake Victoria basin was implemented in March 2014 during the beginning of long rainy season in the basin. As expected mosquito collection in all sentinel villages decreased significantly immediately post-IRS implementation. In Ukerewe, Sengerema and Serengeti districts, anopheline mosquito collection decreased gradually from April to August and collection started to increase steadily from September to December. Generally higher numbers of mosquitoes were collected during the months of November and December in all sentinel villages. These months are within the rainy season but also the residual efficacy of p-methyl 300 CS on mud and painted sprayed wall surfaces had started to fall below the WHOPES recommended threshold of ≥ 80% mosquito mortality. However, unlike in other districts, mosquito abundance in Rorya district could not demonstrate marked patterns with high numbers of mosquitoes being collected throughout the year with both CDC light traps and clay pot traps even immediately post-IRS implementation.

In this study, CSP-ELISA was used to detect presence of sporozoites in malaria vectormosquitoes [60, 61]. The low sporozoite rates observed in this study demonstrates that malaria transmission intensity is low and this might have been contributed by the impact of p-methyl 300 CS IRS and other malaria interventions implemented in the sprayed districts. Four malaria vectors mosquito species were collected and identified in the four districts namely, *An*. *gambiae s*.*s*,*An*. *arabiensis*, *An*. *parensis* and *An*. *rivulorum*. This means that communities in the Lake Victoria basin of Tanzania are exposed to different malaria vectors species. There was significant variation in the abundance of malaria vectors by sentinel village, species, and collection method. Sentinel villages that had relatively higher precipitation such as Bukindo (Ukerewe district) and Katunguru (Sengerema district) had a significantly higher numbers of mosquitoes collected, while Natta-Mbiso sentinel village in Serengeti district had relatively low rainfall and hence fewer mosquitoes collections throughout the monitoring period. This shows clearly that malaria vectors mosquito abundance is a function of rainfall because rainfall produces breeding sites [62, 63].

The PCR results show that principal malaria vectors in the basin are *An*. *gambiae s*.*l*. complex and An. *funestus* group. Traditionally, malaria transmission in much of Tanzania has been dominated by *An*. *gambiae* and *An*. *funestus* which primarily feed and rest indoors where they can be efficiently targeted with insecticides for IRS [64–66]. Pirimiphos methyl 300 CS provides an ideal insecticide for combined use of different classes of insecticides for IRS and LLINs. The use of p-methyl 300 CS would sustain malaria control by delaying the emergence of insecticide resistance especially against organophosphate and pyrethroid insecticides [67]. Pirimiphos-methyl 300 CS is also additionally valuable for controlling pyrethroid-resistant malaria vectors.

There were several limitations to this study. Firstly, this study lacked a control group to better quantify the effect of IRS in comparison with non IRS districts, which prompts us to be cautious about making causal inferences on the estimated effect of the IRS. Secondly, temporal changes in vector density could have been influenced by a number of possible confounding factors which were not controlled for such as environmental factors associated with vector density and changes in the coverage of other malaria control interventions. For example, changes in the use of ITNs were not controlled for in this study. Therefore, decreases in measures of vector density observed in this study could have been partly due to increasing ITNs coverage and not just a result of IRS. Thirdly, another limitation of this study is the use of the sporozoite rate as the outcome measure of malaria decline. Furthermore, the study lacks malaria data to show any impact on malaria incidence and mortality. Fourthly, monthly trends in malaria vector species composition and temporal distribution showed *An*. *gambiae* s.l. to dominate the vector population throughout the year. Two clear peaks of high vector densities in the region were observed to correspond with periods of short rains (October-December) and long rains (March—May). Indoor residual spraying in early March is likely to only be effective against malaria vectors during one of the two malaria transmission peaks following the long rains, which also happens to be the major transmission season. However, the intervention appears unlikely to have much impact against the second minor transmission season that usually follows the short rains in December to February (9–12 months after spraying). Despite these limitations, the overall decline in *Anopheles* mosquitoes in the months following the spraying as well as the subsequent waning of the effect, point to a causal relationship between both variables, as the study team is not aware of any other simultaneous phenomenon or intervention that could explain this behaviour. The mass distribution of LLINs which occurred in 2011 in Tanzania[68] could unlikely explain the findings as similar studies that conducted separate analyses for IRS and LLINs showed that LLINs were not associated with a significant reduction in malaria prevalence while IRS provided a significant added benefit in malaria reduction even in settings were LLINs ownership is high[69]. Despite the study limitations, the results offered draw interest to the effect of IRS on vector density, signifying the reduction of sporozoite rate and/or even elimination are possible following unrelenting and well managed IRS programme.

## Conclusion

The findings of the current study recommend that in Lake Victoria basin, where malaria transmission cycle is biannual, especially where the transmission cycle starts instantly after rainy season, IRS with p-methylin February is probable to only be valuable against malaria vectors during one of the two peak periods following the long rains, which also happens to be the major transmission season. Nonetheless, the intervention appears not likely to have much impact against the second minor transmission season that normally follows the short rains (32–43 weeks post spraying). Furthermore, the finding of this study shows that principal malaria vectors in the Lake Victoria basin of Tanzania are *An*. *gambiae s*.*l*. complex and *An*. *funestus* group. Both *An*. *gambiae* and *An*. *funestus* rest on wall surfaces before and after feeding. Pirimiphos-methyl 300 CS for IRS is therefore effective in killing or repelling major malaria vectors in the study area.

## Supporting information

S1 Table Mosquito collection. (XLS)

S2 Table Bioassay data. (XLS)

S3 Table ELISA PCR results. (XLS)

S4 Table Rainfall data. (XLS)

## Abbreviations

CDC: Centers for Diseases Control
CMCs: Community Mosquito Collectors
CS: Concentrate Suspension
CSP: Circum-Sporozoite Protein
DDT: Dichloro-Diphenyl-Trichloroethane
DITT: District IRS Technical Team
DVCOs: District Vector Control Officers
EC: Emulsifiable Concentrate
ELISA: Enzyme-linked Immunosorbent Assay
IRS: Indoor Residual Spraying
KD: Knockdown
LLINs: Long Lasting Insecticide Treated Nets
LZIRB: Lake Zone Institutional Review Board
NIMR: National Institute for Medical Research
NMCP: National Malaria Control Programme
PCR: Polymerase Chain Reaction
P-methyl: Pirimiphos-methyl
PMI: President’s Malaria Initiative
RH: Relative Humidity
RTI: Research Triangle Institute
SSA: sub-Saharan Africa
THIMS: Tanzania HIV/AIDS and Malaria Indicator Survey
URT: United Republic of Tanzania

## References

[1] Porter JI. Is Art Modern? Kristeller's 'Modern System of the Arts' Reconsidered. British Journal of Aesthetics. 2009;49(1):1–24.

[2] Lengeler C. Insecticide-treated bed nets and curtains for preventing malaria. Cochrane Database Syst Rev. 2004(2):CD000363 Epub 2004/04/24. 10.1002/14651858.CD000363.pub2 15106149

[3] Hawley WA, Phillips-Howard PA, ter Kuile FO, Terlouw DJ, Vulule JM, Ombok M, et al Community-wide effects of permethrin-treated bed nets on child mortality and malaria morbidity in western Kenya. Am J Trop Med Hyg. 2003;68(4 Suppl):121–7. Epub 2003/05/17. 12749495

[4] Hamusse SD, Balcha TT, Belachew T. The impact of indoor residual spraying on malaria incidence in East Shoa Zone, Ethiopia. Global health action. 2012;5:11619 Epub 2012/04/20. 10.3402/gha.v5i0.11619 22514514 PMC3329214

[5] Zhou G, Githeko AK, Minakawa N, Yan G. Community-wide benefits of targeted indoor residual spray for malaria control in the western Kenya highland. Malar J. 2010;9:67 Epub 2010/03/05. 10.1186/1475-2875-9-67 20199674 PMC2843726

[6] Sirima SB, Konate A, Tiono AB, Convelbo N, Cousens S, Pagnoni F. Early treatment of childhood fevers with pre-packaged antimalarial drugs in the home reduces severe malaria morbidity in Burkina Faso. Trop Med Int Health. 2003;8(2):133–9. Epub 2003/02/13. 12581438 10.1046/j.1365-3156.2003.00997.x

[7] Ishengoma DS, Francis F, Mmbando BP, Lusingu JP, Magistrado P, Alifrangis M, et al Accuracy of malaria rapid diagnostic tests in community studies and their impact on treatment of malaria in an area with declining malaria burden in north-eastern Tanzania. Malar J. 2011;10:176 Epub 2011/06/28. 10.1186/1475-2875-10-176 21703016 PMC3145609

[8] Ajayi IO, Browne EN, Bateganya F, Yar D, Happi C, Falade CO, et al Effectiveness of artemisinin-based combination therapy used in the context of home management of malaria: a report from three study sites in sub-Saharan Africa. Malar J. 2008;7:190 Epub 2008/09/30. 10.1186/1475-2875-7-190 18822170 PMC2567328

[9] Ishengoma DS, Mmbando BP, Segeja MD, Alifrangis M, Lemnge MM, Bygbjerg IC. Declining burden of malaria over two decades in a rural community of Muheza district, north-eastern Tanzania. Malar J. 2013;12:338 Epub 2013/09/24. 10.1186/1475-2875-12-338 24053121 PMC3850962

[10] O'Meara WP, Mangeni JN, Steketee R, Greenwood B. Changes in the burden of malaria in sub-Saharan Africa. Lancet Infect Dis. 2010;10(8):545–55. Epub 2010/07/20. 10.1016/S1473-3099(10)70096-7 20637696

[11] Okiro EA, Alegana VA, Noor AM, Snow RW. Changing malaria intervention coverage, transmission and hospitalization in Kenya. Malar J. 2010;9:285 Epub 2010/10/16. 10.1186/1475-2875-9-285 20946689 PMC2972305

[12] Meyrowitsch DW, Pedersen EM, Alifrangis M, Scheike TH, Malecela MN, Magesa SM, et al Is the current decline in malaria burden in sub-Saharan Africa due to a decrease in vector population? Malar J. 2011;10:188 10.1186/1475-2875-10-188 21752273 PMC3160426

[13] Mashauri FM, Kinung'hi SM, Kaatano GM, Magesa SM, Kishamawe C, Mwanga JR, et al Impact of indoor residual spraying of lambda-cyhalothrin on malaria prevalence and anemia in an epidemic-prone district of Muleba, north-western Tanzania. Am J Trop Med Hyg. 2013;88(5):841–9. 10.4269/ajtmh.12-0412 23458959 PMC3752746

[14] Wort UU, Hastings IM, Carlstedt A, Mutabingwa TK, Brabin BJ. Impact of El Nino and malaria on birthweight in two areas of Tanzania with different malaria transmission patterns. Int J Epidemiol. 2004;33(6):1311–9. 10.1093/ije/dyh256 15256522

[15] Kim MJ, Jung BK, Chai JY, Eom KS, Yong TS, Min DY, et al High Malaria Prevalence among Schoolchildren on Kome Island, Tanzania. The Korean journal of parasitology. 2015;53(5):571–4. Epub 2015/11/06. 10.3347/kjp.2015.53.5.571 26537036 PMC4635836

[16] Knox TB, Juma EO, Ochomo EO, Pates Jamet H, Ndungo L, Chege P, et al An online tool for mapping insecticide resistance in major Anopheles vectors of human malaria parasites and review of resistance status for the Afrotropical region. Parasit Vectors. 2014;7:76 10.1186/1756-3305-7-76 24559061 PMC3942210

[17] Kabula B, Tungu P, Malima R, Rowland M, Minja J, Wililo R, et al Distribution and spread of pyrethroid and DDT resistance among the Anopheles gambiae complex in Tanzania. Med Vet Entomol. 2014;28(3):244–52. 10.1111/mve.12036 24192019 PMC10884793

[18] Mmbando AS, Okumu FO, Mgando JP, Sumaye RD, Matowo NS, Madumla E, et al Effects of a new outdoor mosquito control device, the mosquito landing box, on densities and survival of the malaria vector, Anopheles arabiensis, inside controlled semi-field settings. Malar J. 2015;14:494 10.1186/s12936-015-1013-8 26645085 PMC4673850

[19] Wakabi W. Africa counts greater successes against malaria. Lancet. 2007;370(9603):1895–6. 10.1016/S0140-6736(07)61796-6 18074435

[20] Kleinschmidt I, Schwabe C, Shiva M, Segura JL, Sima V, Mabunda SJ, et al Combining indoor residual spraying and insecticide-treated net interventions. Am J Trop Med Hyg. 2009;81(3):519–24. Epub 2009/08/27. 19706925 PMC3836236

[21] Matowo J, Kitau J, Kaaya R, Kavishe R, Wright A, Kisinza W, et al Trends in the selection of insecticide resistance in Anopheles gambiae s.l. mosquitoes in northwest Tanzania during a community randomized trial of longlasting insecticidal nets and indoor residual spraying. Med Vet Entomol. 2015;29(1):51–9. 10.1111/mve.12090 25537754 PMC4359020

[22] Trigg PI, Kondrachine AV. Commentary: malaria control in the 1990s. Bull World Health Organ. 1998;76(1):11–6. 9615492 PMC2305627

[23] Najera JA, Gonzalez-Silva M, Alonso PL. Some lessons for the future from the Global Malaria Eradication Programme (1955–1969). PLoS Med. 2011;8(1):e1000412 10.1371/journal.pmed.1000412 21311585 PMC3026700

[24] WHO. World malaria report.http://www.who.int/malaria/publication/country-profiles/malaria 2009. Accessed on 07th August, 2012 2009.

[25] Whitney JR, Billoski TV, Jones VR. Evidence for Triceratops in Antarctica In: Billoski TV, editor. New Directions in Paleontology. New York: Academic Press; 1997 p. 24–7.

[26] Tia E, Akogbeto M, Koffi A, Toure M, Adja AM, Moussa K, et al [Pyrethroid and DDT resistance of Anopheles gambiae s.s. (Diptera: Culicidae) in five agricultural ecosystems from Cote-d'Ivoire]. Bull Soc Pathol Exot. 2006;99(4):278–82. Situation de la resistance d'anopheles gambiae s.s. (Diptera: Culicidae) aux pyrethrinoides et au DDT dans cinq ecosystemes agricoles de Cote-d'Ivoire. 17111979

[27] Ranson H, N'Guessan R, Lines J, Moiroux N, Nkuni Z, Corbel V. Pyrethroid resistance in African anopheline mosquitoes: what are the implications for malaria control? Trends Parasitol. 2011;27(2):91–8. 10.1016/j.pt.2010.08.004 20843745

[28] Rowland M, Boko P, Odjo A, Asidi A, Akogbeto M, N'Guessan R. A new long-lasting indoor residual formulation of the organophosphate insecticide pirimiphos methyl for prolonged control of pyrethroid-resistant mosquitoes: an experimental hut trial in Benin. PLoS One. 2013;8(7):e69516 10.1371/journal.pone.0069516 23936033 PMC3720653

[29] Tangena JA, Adiamoh M, D'Alessandro U, Jarju L, Jawara M, Jeffries D, et al Alternative treatments for indoor residual spraying for malaria control in a village with pyrethroid- and DDT-resistant vectors in the Gambia. PLoS One. 2013;8(9):e74351 10.1371/journal.pone.0074351 24058551 PMC3772946

[30] WHOPES. Report of the Sixteenth WHOPES Working Group Meeting: WHO/HQ, Geneva; Review of Pirimiphos-methyl 300 CS, Chlorfenapyr 240 SC, Deltamethrin 62.5 SC-PE, Duranet LN, Netprotect LN, Yahe LN, Spinosad 83.3 Monolayer DT, Spinosad 25 Extended Release GR. Geneva: World Health Organization; 2013.

[31] Das M, Srivastava BN, Rao CK, Thapar BR, Sharma GK. Field trial of the effectiveness of indoor-spraying with pirimiphos-methyl emulsion for malaria control in a tribal area of Phulbani district, Orissa State, India. Med Vet Entomol. 1987;1(3):289–95. 2979544 10.1111/j.1365-2915.1987.tb00357.x

[32] Fuseini G, Ebsworth P, Jones S, Knight D. The efficacy of ACTELLIC 50 EC, pirimiphos methyl, for indoor residual spraying in Ahafo, Ghana: area of high vector resistance to pyrethroids and organochlorines. J Med Entomol. 2011;48(2):437–40. 21485386 10.1603/me09286

[33] Kolaczinski K, Kolaczinski J, Kilian A, Meek S. Extension of indoor residual spraying for malaria control into high transmission settings in Africa. 2007 Trans R Soc Trop Med Hyg 101: 852–853. 2007. 10.1016/j.trstmh.2007.04.003 17507065

[34] Nasir SM, Ahmad N, Shah MA, Azam CM. A large-scale evaluation of pirimiphos-methyl 25% WP during 1980–1981 for malaria control in Pakistan. J Trop Med Hyg. 1982;85(6):239–44. 7154146

[35] Frohlich F. Aesthetic Paradoxes of Abstract Expressionism and Pop Art. British Journal of Aesthetics. 1966;6(1):17–25.

[36] Gaiger J. The Philosophy and Politics of Abstract Expressionism. British Journal of Aesthetics. 2001;41(4):455–7.

[37] Gillies MT, Coetzee M. A supplement to the Anophelinae of Africa South of The Sahara (Afrotropical region) Manual, South Africa 1987.

[38] Osborne H. Non-Iconic Abstraction. British Journal of Aesthetics. 1976;16(4):291–304.

[39] WHO: Indoor residual spraying: use of indoor residual spraying for scaling global malaria control and elimination. WHO/HTM/MAL/2006.1112 edn. Geneva: World Health Organisation; 2006.

[40] Holland RA, Kirschvink JL, Doak TG, Wikelski M. Bats use magnetite to detect the earth's magnetic field. PLoS ONE. 2008;3(2):e1676, 1–6. 10.1371/journal.pone.0001676 18301753 PMC2246016

[41] Abbott WS. A method of computing the effectiveness of an insecticide. J Econ Entomol. 1925;18:265–7.

[42] Wirtz RA, Zavala F, Charoenvit Y, Campbell GH, Burkot TR, Schneider I, et al Comparative testing of monoclonal antibodies against Plasmodium falciparum sporozoites for ELISA development. Bull World Health Organ. 1987;65(1):39–45. Epub 1987/01/01. 3555879 PMC2490858

[43] Scott JA, Brogdon WG, Collins FH. Identification of single specimens of the Anopheles gambiae complex by the polymerase chain reaction. Am J Trop Med Hyg. 1993;49(4):520–9. 8214283 10.4269/ajtmh.1993.49.520

[44] Motherwell R, Reinhardt A, editors. Modern Artists in America. New York, New York: Wittenborn Schultz; 1952.

[45] World Heart Federation: RHD in Africa. www.worldheart.org.

[46] Abbott FF. Lights That Are Hidden. Cal West Med. 1925;23(6):758. PMC1654670 18739680

[47] Chiu C, Xian W, Moss CF, editors. Flying in silence: Echolocating bats cease vocalizing to avoid sonar jamming. Proceedings of the National Academy of Sciences of the United States of America; 2008 9 2. 10.1073/pnas.0804408105 PMC2529029 18725624

[48] Chanda E, Chanda J, Kandyata A, Phiri FN, Muzia L, Haque U, et al Efficacy of ACTELLIC 300 CS, pirimiphos methyl, for indoor residual spraying in areas of high vector resistance to pyrethroids and carbamates in Zambia. Journal of medical entomology. 2013;50(6):1275–81. Epub 2014/05/23. 24843932 10.1603/me13041

[49] Haji KA, Thawer NG, Khatib BO, Mcha JH, Rashid A, Ali AS, et al Efficacy, persistence and vector susceptibility to pirimiphos-methyl (Actellic 300CS) insecticide for indoor residual spraying in Zanzibar. Parasites & vectors. 2015;8:628. Epub 2015/12/15. 26652708 10.1186/s13071-015-1239-x PMC4674920

[50] Oxborough RM, Kitau J, Jones R, Feston E, Matowo J, Mosha FW, et al Long-lasting control of Anopheles arabiensis by a single spray application of micro-encapsulated pirimiphos-methyl (Actellic(R) 300 CS). Malar J. 2014;13:37 10.1186/1475-2875-13-37 24476070 PMC3914366

[51] West PA, Protopopoff N, Wright A, Kivaju Z, Tigererwa R, Mosha FW, et al Enhanced protection against malaria by indoor residual spraying in addition to insecticide treated nets: is it dependent on transmission intensity or net usage? PLoS One. 2015;10(3):e0115661 10.1371/journal.pone.0115661 25811379 PMC4374910

[52] Protopopoff N, Van Bortel W, Marcotty T, Van Herp M, Maes P, Baza D, et al Spatial targeted vector control is able to reduce malaria prevalence in the highlands of Burundi. Am J Trop Med Hyg. 2008;79(1):12–8. 18606758

[53] Abilio AP, Kleinschmidt I, Rehman AM, Cuamba N, Ramdeen V, Mthembu DS, et al The emergence of insecticide resistance in central Mozambique and potential threat to the successful indoor residual spraying malaria control programme. Malar J. 2011;10:110 10.1186/1475-2875-10-110 21535872 PMC3096596

[54] Kawada H, Dida GO, Ohashi K, Komagata O, Kasai S, Tomita T, et al Multimodal pyrethroid resistance in malaria vectors, Anopheles gambiae s.s., Anopheles arabiensis, and Anopheles funestus s.s. in western Kenya. PLoS One. 2011;6(8):e22574 10.1371/journal.pone.0022574 21853038 PMC3154902

[55] Mathias DK, Ochomo E, Atieli F, Ombok M, Bayoh MN, Olang G, et al Spatial and temporal variation in the kdr allele L1014S in Anopheles gambiae s.s. and phenotypic variability in susceptibility to insecticides in Western Kenya. Malar J. 2011;10:10 10.1186/1475-2875-10-10 21235783 PMC3029224

[56] Kabula B, Tungu P, Matowo J, Kitau J, Mweya C, Emidi B, et al Susceptibility status of malaria vectors to insecticides commonly used for malaria control in Tanzania. Trop Med Int Health. 2012;17(6):742–50. 10.1111/j.1365-3156.2012.02986.x 22519840

[57] Damien GB, Djenontin A, Rogier C, Corbel V, Bangana SB, Chandre F, et al Malaria infection and disease in an area with pyrethroid-resistant vectors in southern Benin. Malar J. 2010;9:380 10.1186/1475-2875-9-380 21194470 PMC3224346

[58] Rowland M, Hewitt S, Durrani N, Saleh P, Bouma M, Sondorp E. Sustainability of pyrethroid-impregnated bednets for malaria control in Afghan communities. Bull World Health Organ. 1997;75(1):23–9. 9141747 PMC2486983

[59] Ngufor C, N'Guessan R, Boko P, Odjo A, Vigninou E, Asidi A, et al Combining indoor residual spraying with chlorfenapyr and long-lasting insecticidal bed nets for improved control of pyrethroid-resistant Anopheles gambiae: an experimental hut trial in Benin. Malar J. 2011;10:343 10.1186/1475-2875-10-343 22087506 PMC3229591

[60] Beier JC, Perkins PV, Koros JK, Onyango FK, Gargan TP, Wirtz RA, et al Malaria sporozoite detection by dissection and ELISA to assess infectivity of afrotropical Anopheles (Diptera: Culicidae). J Med Entomol. 1990;27(3):377–84. 2185363 10.1093/jmedent/27.3.377

[61] Burkot TR, Williams JL, Schneider I. Identification of Plasmodium falciparum-infected mosquitoes by a double antibody enzyme-linked immunosorbent assay. Am J Trop Med Hyg. 1984;33(5):783–8. 6385740 10.4269/ajtmh.1984.33.783

[62] Muturi EJ, Shililu J, Jacob B, Gu W, Githure J, Novak R. Mosquito species diversity and abundance in relation to land use in a riceland agroecosystem in Mwea, Kenya. J Vector Ecol. 2006;31(1):129–37. 16859101 10.3376/1081-1710(2006)31[129:msdaai]2.0.co;2

[63] Mwangangi JM, Shililu J, Muturi EJ, Muriu S, Jacob B, Kabiru EW, et al Anopheles larval abundance and diversity in three rice agro-village complexes Mwea irrigation scheme, central Kenya. Malar J. 2010;9:228 10.1186/1475-2875-9-228 20691120 PMC2927610

[64] Russell TL, Beebe NW, Bugoro H, Apairamo A, Chow WK, Cooper RD, et al Frequent blood feeding enables insecticide-treated nets to reduce transmission by mosquitoes that bite predominately outdoors. Malar J. 2016;15:156 Epub 2016/03/13. 10.1186/s12936-016-1195-8 26969430 PMC4788858

[65] Seyoum A, Sikaala CH, Chanda J, Chinula D, Ntamatungiro AJ, Hawela M, et al Human exposure to anopheline mosquitoes occurs primarily indoors, even for users of insecticide-treated nets in Luangwa Valley, South-east Zambia. Parasit Vectors. 2012;5:101 Epub 2012/06/01. 10.1186/1756-3305-5-101 22647493 PMC3432592

[66] White GB. Anopheles gambiae complex and disease transmission in Africa. Trans R Soc Trop Med Hyg. 1974;68(4):278–301. 4420769 10.1016/0035-9203(74)90035-2

[67] N'Guessan R, Rowland M. Indoor residual spraying for prevention of malaria. Lancet Infect Dis. 2012;12(8):581–2. 10.1016/S1473-3099(12)70139-1 22682535

[68] Eze IC, Kramer K, Msengwa A, Mandike R, Lengeler C. Mass distribution of free insecticide-treated nets do not interfere with continuous net distribution in Tanzania. Malar J. 2014;13:196 Epub 2014/06/03. 10.1186/1475-2875-13-196 24884786 PMC4046070

[69] West PA, Protopopoff N, Wright A, Kivaju Z, Tigererwa R, Mosha FW, et al Indoor residual spraying in combination with insecticide-treated nets compared to insecticide-treated nets alone for protection against malaria: a cluster randomised trial in Tanzania. PLoS Med. 2014;11(4):e1001630 Epub 2014/04/17. 10.1371/journal.pmed.1001630 24736370 PMC3988001

